# Anti-Proliferative and Pro-Apoptotic Effects of Digested Aglianico Grape Pomace Extract in Human Colorectal Cancer Cells

**DOI:** 10.3390/molecules27206791

**Published:** 2022-10-11

**Authors:** Giusy Rita Caponio, Miriam Cofano, Tamara Lippolis, Isabella Gigante, Valentina De Nunzio, Graziana Difonzo, Mirella Noviello, Luigi Tarricone, Giuseppe Gambacorta, Gianluigi Giannelli, Maria De Angelis, Maria Notarnicola

**Affiliations:** 1 National Institute of Gastroenterology “Saverio de Bellis”, Research Hospital, Castellana Grotte, 70013 Bari, Italy; 2 Department of Soil, Plant and Food Sciences, University of Bari Aldo Moro, Via Amendola 165/A, 70126 Bari, Italy; 3 Council for Agricultural Research and Economics (CREA), Research Center for Viticulture and Enology, Via Casamassima 148, 70010 Bari, Italy

**Keywords:** antioxidants, antiradical scavenging activity, apoptosis, colorectal cancer, polyphenols

## Abstract

Grape pomace (GP)—the major by-product of winemaking processes—still contains bioactive molecules with known beneficial properties for human health, such as an antiradical scavenging activity or an antiproliferative activity of tumors. In vitro studies have demonstrated that GP polyphenols specifically influence colon cancer cell proliferation. In addition to previously published work, we tested the phenolic compounds of Aglianico GP following an in vitro simulated gastrointestinal digestion on colorectal cancer cell lines at different degrees of differentiation. Our experiments, using HT29 and SW480 cells, confirmed the anti-proliferative effect of GP gastrointestinal digested extract and provided intriguing insights on the way it influences the cancer cell features (i.e., viability, proliferation, and apoptosis). We observed that Aglianico GP extract showed a great ability to affect cell proliferation and apoptosis. Interestingly, both HT29 and SW480 cells produced a significant increase in Bax, and a significant increase in the Bax/Bcl-2 ratio and caspase-3. The gastrointestinal digested GP extract was previously characterized both for antioxidant activity and phenolic composition. As a result, the TPC and the antioxidant activity reached high values in the Aglianico GP digested extract, and the main compounds assessed by UHPLC-DAD were anthocyanins, phenolic acids, and flavonoids. This work shed light on the use of digested GP extract as a dietary ingredient, a very sustainable source of nutritional compounds with potential health benefits for colon cancer cell proliferation.

## 1. Introduction

In the context of research interest on the nutritional value of foods and the effects of polyphenols on human health, in recent years the recovery of polyphenols also from by-products of the main agri-food industries has gained increasing importance [1]. In this scenario, grape pomace (GP)—the major by-product of winemaking processes—is intensively investigated for its beneficial and healthful properties. In fact, the enhancement of the by-products of winemaking is a growing theme not only in the agri-food sector, but recently it has also involved other sectors of different interests such as the biomedical and nutraceutical sectors [2,3]. The beneficial properties mentioned above are mainly attributable to the soluble dietary fibers and polyphenolic component of GP. In particular, among the polyphenolic component of GP, the main bioactive molecules are represented by anthocyanins, phenolic acids, and flavonoids, whose specific biological functions are reported [4].

Specifically, catechins, resveratrol, gallic acid, and other natural compounds [5,6] are known to exert antiradical scavenging activity, antiproliferative activity of tumors, and to protect membranes from oxidation [7]. Data obtained in animal and cellular models confirmed that polyphenols act as antioxidants, neutralizing the free radicals responsible for aging, with anticancer effects [8,9,10].

A rapidly growing body of evidence supports the crucial role of cancer therapy in identifying compounds that induce cells apoptosis, performing a pivotal role in eliminating mutated hyperproliferating cells from the system. Thus, the induction of apoptosis in cancer cells is a favorable protective mechanism against cancer development and progression [11]. Polyphenols are involved in anti-proliferative and antineoplastic activities by influencing proliferation, differentiation, and apoptosis in several types of cancer cells, including colon cancer [12]. Interestingly, an essential aspect of the chemopreventive action of polyphenols consists in their differential activity in targeting cancer cells but not normal cells [13,14]. D’Angelo et al. [11] demonstrated that several polyphenolic compounds are able to modulate cell growth by inducing cell cycle blocking in human cancer cell lines.

Resveratrol—a predominant polyphenol found in grapes—exhibits a potential health benefit in the prevention and treatment of human cancer, according to clinical studies [15,16]. Moreover, it was observed that resveratrol inhibited the growth of DU145 cells of human prostate cancer and provided a molecular explanation for the effect, as treatment with resveratrol in the above-mentioned cells resulted in a dose-dependent inhibition of cell growth and induced cell death by apoptosis [17]. Altogether, the following study suggests that resveratrol has strong development potential as an agent for the prevention of human prostate cancer. In addition, grape polyphenols extracts highlighted a strong ability to affect cell proliferation and apoptosis in the Caco2 cell line and to reduce the pERK/totERK ration protein expression in a dose-dependent manner, as previously shown [18]. Particularly, the mechanisms of mitochondrial apoptosis are linked to the presence of B-cell lymphoma-2 (Bcl-2) family proteins, mainly including the pro-apoptotic Bax protein and the anti-apoptotic Bcl-2 protein [19].

Of note, several aspects influence the real beneficial effects of the intake of polyphenols on the human body. One of these is represented by the concept of bioavailability of polyphenols, which in turn depends on the relative content of compounds released from the food matrix along the digestive system (bioaccessibility), the digestive stability, and the efficiency of the transepithelial passage (intestinal absorption) [20,21,22]. As previously published, the in vitro simulated gastrointestinal digestion of GP extracts improved the bioavailability and bioaccessibility of polyphenols with a consequent increase in both antioxidant activity and phenolic compound content [23]. A useful option to evaluate the bioactivity effects of these extracts consists in using cell lines that reproduce in vitro the mechanisms of action in vivo. Among cell cultures, HT29 are among those of advanced colon cancer. In fact, research is mainly based on studies on HT29 and SW480 cells, and this may be due to the fact that mutations and phenotypic characteristics are representative of most colorectal tumors [24]. In addition, to the best of our knowledge, there are no studies in the literature that have tested the in vitro gastrointestinal digested Aglianico GP extract on cancer cells evaluating the real effects of polyphenols after simulated gastrointestinal digestion. In this scenario, we aimed to evaluate the mechanisms of apoptosis on two lines of colorectal cancer cells at different degrees of differentiation following exposure to gastrointestinal digested GP extract. In particular, we tested how the Aglianico GP gastrointestinal digested extract, at different concentrations, is involved in colon cancerogenesis or cancer progression and the way it influences the cancer cell features (i.e., viability, proliferation, and apoptosis). The gastrointestinal digested GP extract was previously characterized both for antioxidant activity and phenolic composition.

## 2. Results and Discussion

### 2.1. Chemical Characterization of Aglianico GP Gastrointestinal Digested Extract—Antioxidant Activity and Phenolic Composition

Several studies have demonstrated that the in vitro gastrointestinal digestion increased the bioaccessibility of polyphenols [23,25]. Thus, in this study we determined the antioxidant activity, the total phenolic content (TPC), and the polyphenolic composition of the GP gastrointestinal digested extract. The phenolic composition of the Aglianico GP gastrointestinal digested extract, as well as the antioxidant activity, is detailed in Table 1. Notably, the Aglianico GP gastrointestinal digested extract reached high values of antioxidant activity by ABTS and DPPH assays, 36.00 ± 0.24 µmol TE/dry weight and 15.18 ± 0.10, respectively. Moreover, the TPC evaluated according to the Folin–Ciocalteu method was 5.01 ± 0.16 mg GA/DW. 

Table 2 shows the polyphenolic composition of the Aglianico GP gastrointestinal digested extract. The content of quantified phenolic compounds by UHPLC-DAD analysis was listed in Table 2. The main detected phenolic compounds were divided into anthocyanins, phenolic acids, and flavonoids classes. As shown, the greater contribution of the total polyphenols was derived from anthocyanins, especially malvidin-3-*O*-glucoside (184.5 ± 19.3 mg/kg dry weight) and malvidin-3-*O*-p-coumaroylglucoside (148.7 ± 1.7 mg/kg dry weight). Moreover, gallic acid represented a considerable amount of the total, with 189.2 ± 4.9, and among flavonoids rutin hydrate was detected. Overall, these results, both in terms of antioxidant activity and polyphenols content, are in line with previously reported work [23], which conducted a study on GP of the same variety of Aglianico.

### 2.2. Effects of Aglianico GP Gastrointestinal Digested Extract on Cell Growth

**Figure 1:**
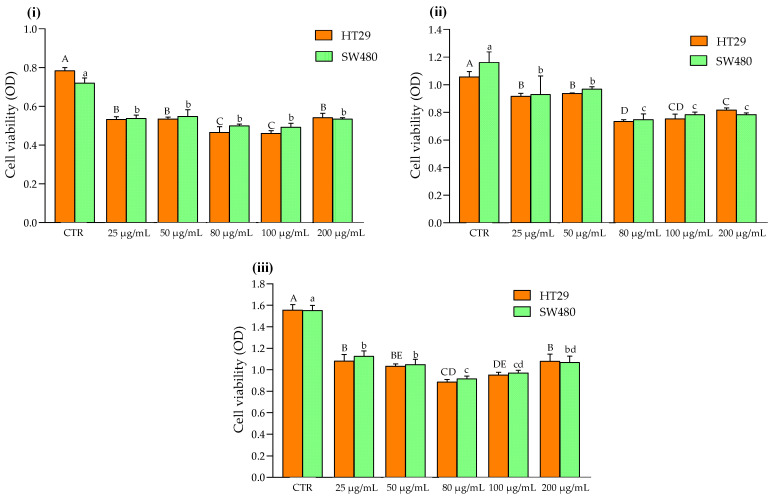
Effects of Aglianico GP gastrointestinal digested extract on cell proliferation of HT29 and SW480 cells treated with increasing concentrations. (**i**) Cell viability of cells after 24 h; (**ii**) Cell viability of cells after 48 h; (**iii**) Cell viability of cells after 72 h. All data are the means ± SD of three replicate measurements. Values are expressed as cell viability (optical density (OD) at 570 nm). Different capital letters (A–E) indicate a significant difference (*p* < 0.05, one-way ANOVA, and Tukey’s HSD test) when comparing different GP concentrations in HT29 cell line; different lower-case letters (a–d) indicate a significant difference (*p* < 0.05, one-way ANOVA, and Tukey’s HSD test) when comparing different GP concentrations in SW480 cell line.

The effects of the Aglianico GP gastrointestinal digested extract on cell proliferation were evaluated by the MTT assay. As shown in Figure 1, both HT29 and SW480 cells were inhibited by exposure at increasing polyphenol extracts. Specifically, 25 μg/mL of Aglianico GP gastrointestinal digested extract was already able to exert significant antiproliferative effects on SW480 and HT29 cells after 24 h of incubation (Figure 1, panel (i)). Regarding SW480 cells, the inhibition of cell proliferation did not take place in a dose-dependent manner. In fact, 25, 50, 80, 100, and 200 μg/mL of polyphenols highlighted a significant reduction in cell proliferation compared to the CTR (0 μg/mL), without significant differences among different concentrations (Figure 1, panel (i)). Instead, for HT29 cells, 25 and 50 μg/mL of polyphenols reached a significant reduction in cell proliferation (0.53 ± 0.01 and 0.53 ± 0.01, respectively) compared to the CTR (0.78 ± 0.02). Nevertheless, a further significant decrease was recorded at 80 and 100 μg/mL of Aglianico GP gastrointestinal digested extract on cell proliferation. Then, a slight increase in cell proliferation was found for 200 μg/mL of polyphenols extract.

The antiproliferative action exerted by GP polyphenols was also observed on HT29 and SW480 cells after 48 h of incubation (Figure 1, panel (ii)) and 72 h of incubation (Figure 1, panel (iii)). A similar trend was also found for samples after 48 h of incubation. Specifically, SW480 cells showed a lower cell proliferation at 80 and 100 μg/mL of polyphenols extract, followed by 25 and 50 μg/mL. Similarly, the polyphenols extract inhibited the HT29 cell proliferation, with a significant decrease at 80 μg/mL (0.74 ± 0.01). Previous scientific studies reported that HT29 cells exposed to an increasing concentration of proanthocyanins inhibited the cell proliferation after 24 h of treatment [26]. Moreover, pomegranate polyphenols have also been demonstrated to decrease cell proliferation on colon rectal cancer cells by inducing apoptosis [27]. Considering that the concentration of 25 μg/mL of polyphenols extract was already able to exert a significant antiproliferative effect on HT29 and SW480 cells after 24 h, these samples were chosen to investigate the expression of some proteins and genes involved in cell proliferation and apoptosis.

### 2.3. Pro-Apoptotic Activity of Aglianico GP Gastrointestinal Digested Extract

**Figure 2:**
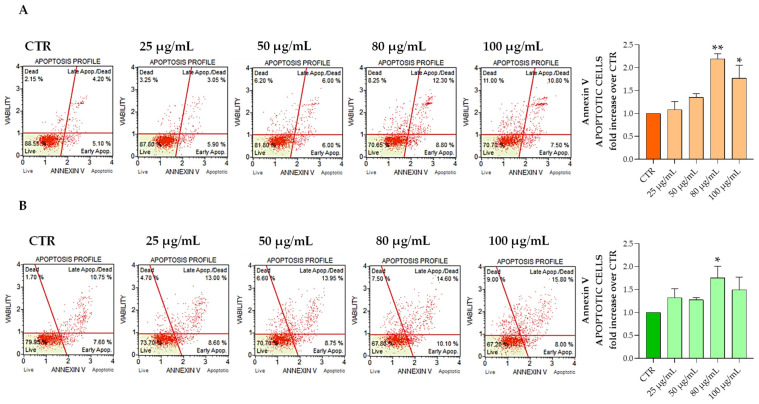
HT29 and SW480 cells cultured with Aglianico GP gastrointestinal digested extract at increasing concentrations for 24 h, processed and analyzed for the percentage of live, early/ late apoptotic, and dead cells by Muse Annexin V assay. The panels represent an example of apoptosis profile in different treatment conditions for HT29 (**A**) and SW480 (**B**), respectively. The mean of three independent experiments of apoptotic cells was plotted in the relative graph. The results are expressed as means ± SD. * *p* < 0.01; ** *p* < 0.001.

Since cell growth is a balance between proliferation and apoptosis, the percentage of apoptotic cells was then examined after a treatment with the Aglianico GP gastrointestinal digested extract at increasing concentrations for 24 h compared with that of the control. The obtained results show that the Aglianico GP gastrointestinal digested extract was able to increase the apoptotic cells both in the HT29 and SW480 cell lines (Figure 2). Specifically, apoptotic cells significantly increased at 80 μg/mL and 100 μg/mL in the HT29, and at 80 μg/mL in the SW480 cell line. These results were also confirmed by Hoechst 33342 staining experiments (Figure S1).

Thus, when Aglianico GP extract was added to the HT29 cell medium, a pronounced and significant apoptosis induction was found (Figure 2). Annexin V assay was used to determine the apoptotic cell population. Apoptosis is a well-controlled process of cell elimination with a pivotal role in the regulation of physiological growth control and homeostasis. Insufficient apoptosis plays a key role in tumor progression and resistance to therapies [28]. More precisely, in HT29, the apoptotic cells achieved higher values, with 21.35 ± 0.35 at 80 μg/mL and 17.16 ± 1.59 in 100 μg/mL, compared to the control (9.78 ± 0.67). These data, in accordance with the main results obtained by Suganya et al. [29], indicated a significant increase in cell death in a dose-dependent manner, suggesting that GP induces apoptosis in HT29 cells.

In addition, the Aglianico GP gastrointestinal extract on the SW480 cells produced a significant apoptotic effect, particularly at a concentration of 80 μg/mL (28.7 ± 0.14), compared to the control (16.60 ± 2.51). Our results are in line with other studies that found a pronounced increase in apoptosis in SW480 cells treated with 50 mg/L of grape seed procyanidins, approximately 30% compared to the control [30]. Moreover, recent literature demonstrated that some flavonoids from grapes and hops induced apoptosis in SW480 using the annexin V assay [31,32].

**Figure 3:**
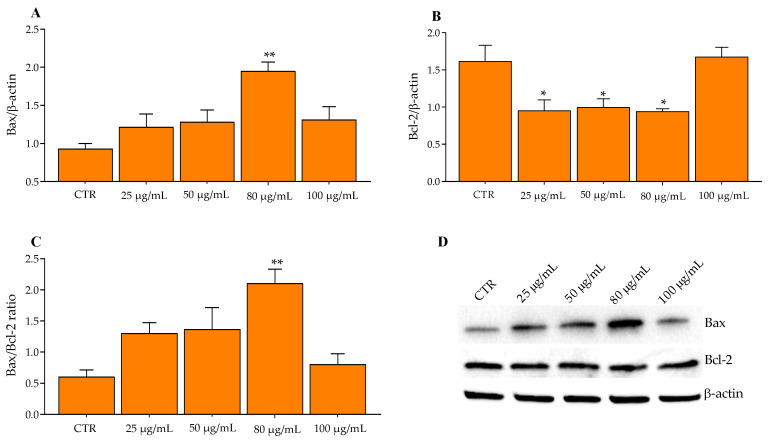
Western blotting analysis of proteins in HT29 cells exposed with Aglianico GP gastrointestinal digested extract at increasing concentrations for 24 h. (**A**) Bax proteins; (**B**) Bcl-2 proteins; (**C**) Bax/Bcl-2 ratio; (**D**) Relative blots. All data represent the result of three different experiments (means ± SD). *p*-value was determined by ANOVA with Dunnett’s test. * *p* < 0.01 and ** *p* < 0.001.

Tacking in account the above-mentioned information, Bax, Bcl-2, Caspase-3, and Caspase-8 protein expressions were measured in HT29 and SW480 cells by Wester blotting after the incubation of increasing concentrations of Aglianico GP gastrointestinal digested extract (25, 50, 80, 100 μg/mL) for 24 h. The proteins of the Bcl-2 family play an important role in the mitochondrial apoptotic pathway following the exposure of HT29 cells to extracts. In fact, as shown in Figure 3A, compared to the CTR (0 μg/mL), a significant increase in Bax after treatment with Aglianico GP gastrointestinal digested extracts at 80 μg/mL was observed, as well as a significant decrease in Bcl-2 already at lower concentrations (25, 50 μg/mL) (Figure 3B). Consequently, the exposure of HT29 cells treated with extracts resulted in a significant increase in the Bax/Bcl2 ratio (Figure 3C) at the concentration of 80 μg/mL, but decreased reaching values similar to CTR, at 100 μg/mL. These results are in line with a previous study which established that a chrysin derivative—a type of flavonoid—induced the mitochondrial apoptotic pathway in HT29 cells through the regulation of Bcl-2 family proteins and Caspases-9 and Caspase-3. In fact, the exposure of flavonoid for 12 h significantly increased the levels of the pro-apoptotic protein (Bax) and decreased those of the anti-apoptotic protein (Bcl-2) at concentrations of 50 and 100 μg/mL in HT29 cells [33]. Similar results were found following the exposure of HT29 cells to other types of flavonols—hyperoside and rutin—abundant in wine, grapes, and citrus fruits [34], showing an increase in Bax expression, a reduction in Bcl-2, and an increase in Bax/Bcl-2 in a dose-dependent manner [35]. Furthermore, our results were also supported by a recent study performed on HT29, MCF-7, and PC-3 cells treated with proanthocyanidins for 24 h and 48 h [26], highlighting a significant increase and decrease in Bax and Bcl-2, respectively. In addition, Reddivari et al. [36] have shown that a bioactive compound of grapes, in particular resveratrol, together with grape seed extract, induced apoptosis in colon cancer stem cells. The main result of this study (a significant increase in the Bax/Bcl-2 ratio), demonstrates how the combination of the polyphenol mixture displays an antitumor effect with results similar to a chemotherapy drug used as a control. 

**Figure 4:**
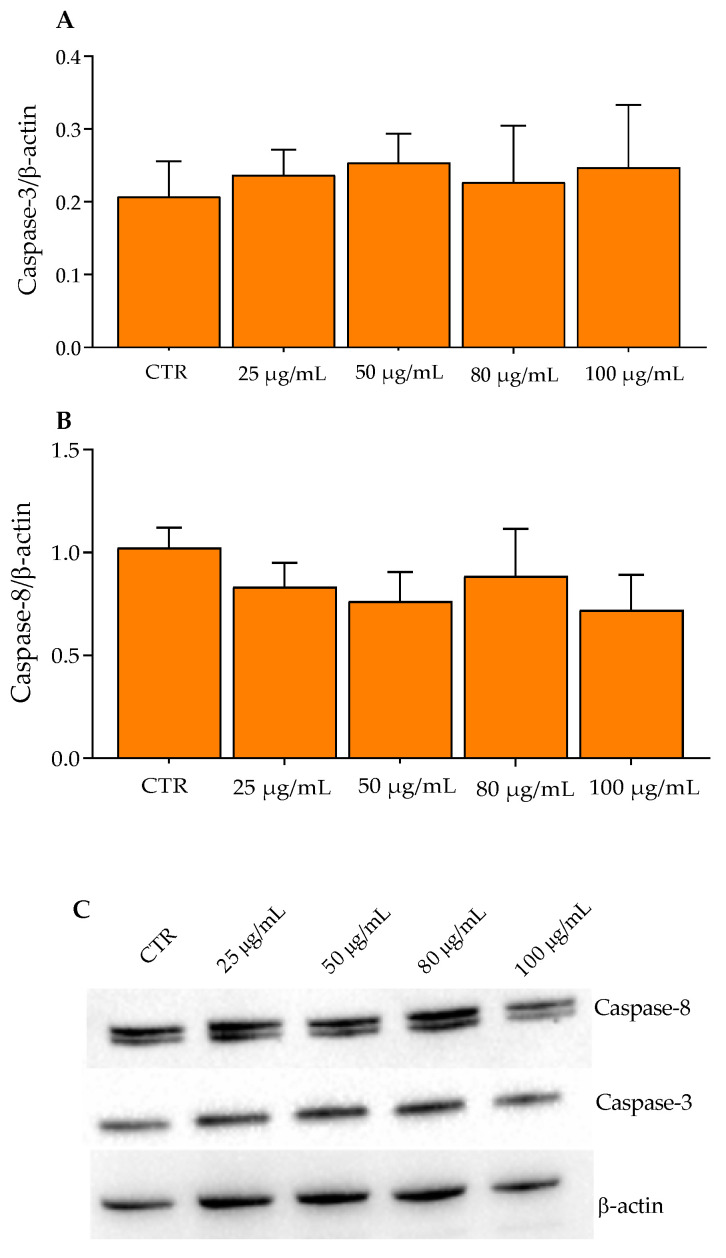
Western blotting analysis of Caspase proteins in HT29 cells exposed to Aglianico GP gastrointestinal digested extract at increasing concentrations for 24 h. (**A**) Caspase-3 proteins; (**B**) Caspase-8 proteins; (**C**) Relative blots. All data represent the results of three different experiments (means ± SD).

Of note, GP polyphenols activate the apoptosis through the induction of the mitochondrial apoptotic pathway, in particular by reducing the anti-apoptotic protein Bcl-2 and increasing the pro-apoptotic Bax. Notably, anti-apoptotic proteins—associated with the mitochondrial outer membrane—are responsible for the integrity of mitochondria (Figure S2). Following an apoptotic stimulus, pro-apoptotic proteins such as Bax and Bak oligomerize in the mitochondrial outer membrane, causing changes in its integrity by the formation of membrane pores with the consequent release of cytochrome C and the activation of caspases [35,37,38]. If on the one hand GP had activated the Bax pathway of apoptosis, on the other hand no influence was observed for the Caspase pathway. In fact, HT29 cells treated with increasing concentrations of Aglianico GP gastrointestinal digested extracts showed a similar trend both for Caspase-3 and Caspase-8 compared to the control (Figure 4A,B).

**Figure 5:**
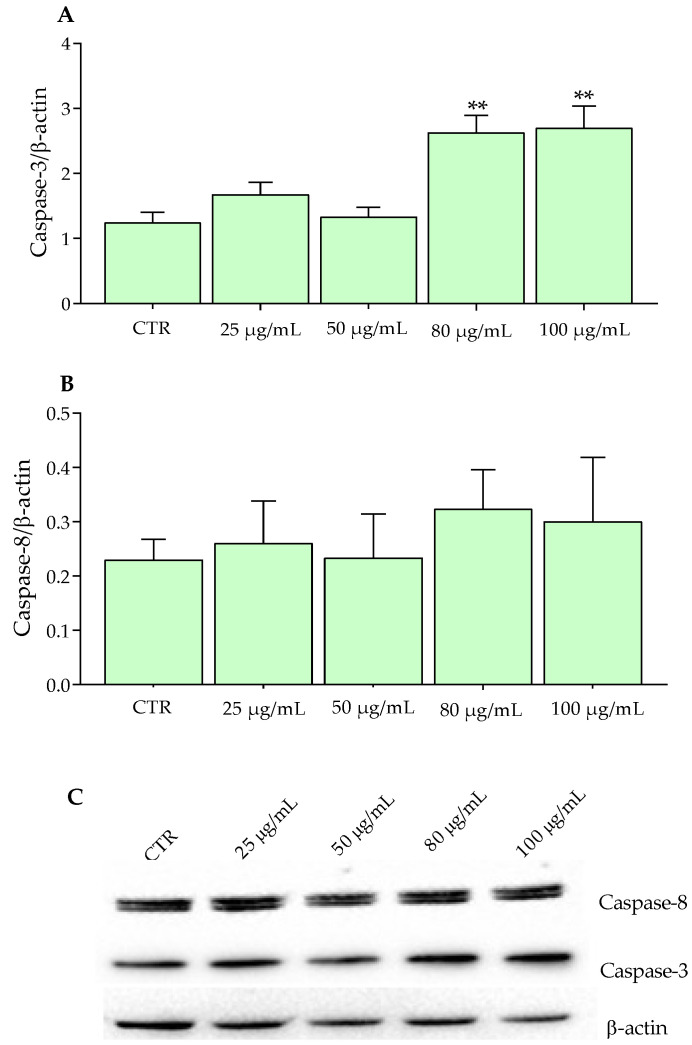
Western blotting analysis of Caspase proteins in SW480 cells exposed to Aglianico GP gastrointestinal digested extract at increasing concentrations for 24 h. (**A**) Caspase-3 proteins; (**B**) Caspase-8 proteins; (**C**) Relative blots. All data represent the result of three different experiments (means ± SD). *p*-value was determined by ANOVA with Dunnett’s test. ** *p* < 0.001.

On the contrary, SW480 cells exhibited a significant increase in Caspase-3 at concentrations of 80 and 100 μg/mL after 24 h of incubation (Figure 5A). In fact, as reported by Sithara et al. [39], the exposure of SW480 at different concentrations (150, 200, and 250 μM) of morin flavonoid for 48 h determined a significant increase in Caspase-3 involved in the apoptosis mechanisms of cells. Moreover, not just the exposure to a single flavonoid, but a combination of these, exerted beneficial effects on the cancer cells; in fact, in a recent work, the combination of two flavones, namely apigenin (25 μm) and chrysin (25 μm), determined an increase in the protein expression of Caspase 3, compared to the single administration, evaluated in SW480 cells [40]. 

**Figure 6:**
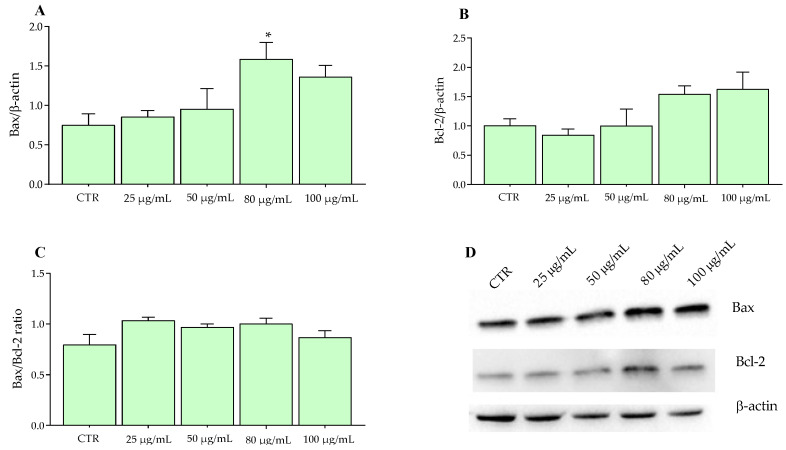
Western blotting analysis of proteins in SW480 cells exposed to Aglianico GP gastrointestinal digested extract at increasing concentrations for 24 h. (**A**) Bax proteins; (**B**) Bcl-2 proteins; (**C**) Bax/Bcl-2 ratio; (**D**) Relative blots. All data represent the results of three different experiments (means ± SD). *p*-value was determined by ANOVA with Dunnett’s test. * *p* < 0.01.

As confirmed by MTT results on cell proliferation, already at lower concentrations, certain polyphenols expressed their pro-apoptotic activity. In this context, 10–20 μM of geraniin significantly reduced the expression of Caspase-3 [41]. Similar findings were reported by Hsu et al. [30] in SW480 cells treated for 24 h with procyanidins contained in grape seeds. Figure 6A shows a significant increase in Bax at a concentration of 80 μg/mL in SW480 cells, in accordance with the above-mentioned [30,39,40,41]. 

Although the two cell lines belong to colorectal cancer lines, they differ in both morphology and degree of differentiation (i.e., HT29 is more differentiated than SW480) [42,43,44]. Additionally, scientific studies highlighted a distinct molecular background and different expression of genes associated with proliferation/anti-proliferation, as well as DNA repair, in the above-mentioned cells [45,46]. Thus, an increased and/or decreased expression in Bax, Bcl-2, and Caspase-3 and -8 is probably correlated with the different characteristics concerning the morphological and genetical aspects as well as degrees of differentiation of HT29 and SW480. As confirmed by our results, previous studies showed different expressions of both apoptotic rate and apoptotic markers in these cells [30,41,47].

## 3. Materials and Methods

### 3.1. Chemicals and Reagents

Ethanol for residual analysis—acetonitrile HPLC grade—was purchased from Sigma-Aldrich (St. Louis, MO, USA) and formic acid HPLC grade from Muskegon (Muskegon, MI, USA); ABTS (2,20-azino-bis(3-ethylbenzothiazoline-6-sulphonic acid) diammonium salt) DPPH (2,2-diphenyl-1-picrylhydrazyl), gallic acid, and syringic acid were purchased from Sigma-Aldrich (Darmstadt, Germany); α-amylase, pepsin, and pancreatin were purchased from Sigma-Aldrich Chemistry (St. Louis, MO, USA); and bile salts were purchased from Oxoid (Hampshire, UK). Malvidin-3-*O*-glucoside and rutin hydrate were obtained from phyproof^®^ (PhytoLab, Dutendorfer, Germany). Roswell Park Memorial Institute (RPMI), Dulbecco’s Modified Eagle’s Medium (DMEM), fetal bovine serum (FBS), phosphate-buffered saline (PBS), and penicillin were purchased from Sigma-Aldrich (Milan, Italy). HT29 and SW480 human colon cancer cell lines were purchased from the National Institute of Biomedical Innovation JCRB Cell Bank (Osaka, Japan).

### 3.2. Aglianico GP Sampling and Phenol Extract Preparation

The research—conducted in December 2021—was based on Aglianico grape cultivars from the vineyard in the Corato area (Apulia Region, Southern Italy). The GP cultivar of Aglianico was obtained as a by-product of red winemaking processes carried out at an experimental vinery of the Department of Soil, Plant and Food Sciences (Di.S.S.P.A), University of Bari Aldo Moro. GP underwent 5 days of maceration at 25 °C, 2 punching-down per day, with the addition of potassium metabisulphite (6 g/hL), yeast (*Saccharomyces cerevisiae* var. Bayanus, LALVIN R2™, 20 g/hL), and yeast activator. GP was used for the extraction of phenolic compounds by using a water extraction according to Caponio et al. [23]. Briefly, GP was mixed with distilled water (1:2, *w*/*v*) and submitted to intense agitation with a stomacher 400 lab blender (Seward Medical, London, UK) for 180 s. The extracts were recovered by filtration using filter paper (Cordenons, PN, Italy), followed by a nylon filter (pore size 0.45 mm, Sigma, Ireland), and then stored at −20 °C until analysis.

### 3.3. In Vitro Gastrointestinal Digestion of the Aglianico GP Extract

The Aglianico GP extract followed the in vitro gastrointestinal digestion according to the method described by Kamiloglu and Capanoglu [48], with slight modifications. The in vitro gastrointestinal digestion was performed, comprising a pepsin–HCl digestion for 3 h at 37 °C (to simulate gastric digestion) and a pancreatin digestion with pancreatin and bile salts for 3 h at 37 °C (to simulate small intestinal digestion).

Briefly, 10 mL of extract was added to α-amylase (56 mg/mL) (Sigma-Aldrich Chemistry, St. Louis, MO, USA) and to 10 mL of pepsin solution composed by NaCl 125 mM/L + KCl 7 mM/L + NaHCO_3_ 45 mM/L + pepsin (Sigma-Aldrich Chemistry, St. Louis, MO, USA) 3 g/L. Thus, the pH was adjusted to 2, using HCl, and incubated at 37 °C for 180 min in a water bath under shaking. After incubation, was added in equal volume an intestinal solution. The intestinal solution was simulated by dissolving 0.1 g/100 mL of pancreatin (Sigma-Aldrich Chemistry, St. Louis, MO, USA) and 0.15 g/100 mL of bile salts (Oxoid™, Hampshire, UK). The pH was adjusted to 8, using NaOH, and incubated at 37 °C for 180 min in a water bath under shaking. After incubation, an aliquot of intestinal-digested extract was kept and stored at −20 °C before analysis. Extracts were then filtered using 0.45 μm Whatman filter paper and then analyzed for antioxidant activity and quantitative UHPLC-DAD analysis of phenolic compounds. Moreover, a sample without Aglianico GP extract (containing 0 μg/mL of polyphenols), that followed the same gastrointestinal digestion condition, was used as a control (CTR) in the following treatment on cell cultures.

### 3.4. Determination of Total Phenol Content and Antioxidant Activity

Aglianico GP gastrointestinal digested extract was evaluated for the TPC according to the Folin–Ciocalteu method, as reported in Caponio et al. [49]. Briefly, to 980 μL of H_2_O Milli-Q, 20 μL of appropriately diluted extract and 100 μL of Folin–Ciocalteu reagent were added. After 3 min, 800 μL of 7.5% Na_2_CO_3_ were added and then the sample was stored in the dark for 60 min. The absorbance was read at 720 nm using an Evolution 60s UV-visible spectrophotometer (Thermo Fisher Scientific, Rodano, Italy). The results were expressed as mg of gallic acid equivalents (GAEs) per g of dry weight (DW) sample (mg GAE/g DW). Each sample was analyzed in duplicate. The antioxidant activities of the GP extracts were measured by ABTS and DPPH assays, as reported by Caponio et al. [49]. The DPPH (2,2-diphenyl-1-picrylhydrazyl) assay was performed by preparing a solution of DPPH 0.08 mM in ethanol. In cuvettes for spectrophotometry, 50 µL of each sample was added to 950 µL of DPPH solution. After 30 min in the dark, the decrease in absorbance was measured at 517 nm using an Evolution 60s UV-visible spectrophotometer (Thermo Fisher Scientific, Rodano, Italy).

The ABTS [2,2′-azino-bis(3-ethylbenzothiazoline-6-sulfonic acid)] radical was generated by a chemical reaction with potassium persulfate (K_2_S_2_O_8_). Briefly, 25 mL of ABTS (7 mM in H_2_O) was spiked with 440 μL of K_2_S_2_O_8_ (140 mM) and kept in the dark at room temperature for 12–16 h. The working solution, by diluting with H_2_O, was prepared to obtain a final absorbance at 734 nm equal to 0.80 ± 0.02 [50]. The decrease in absorbance was measured at 734 nm after 8 min of incubation. Results were expressed as μmol Trolox equivalents (TE)/g of DW. Each sample was analyzed in triplicate.

### 3.5. UHPLC-DAD Phenolic Analysis of Aglianico GP Gastrointestinal Digested Extract

The phenol composition of Aglianico GP gastrointestinal digested extract was determined by UHPLC Ultimate 3000 RS Dionex system (Thermo Fisher Scientific, Waltham, MA, USA) composed by LPG-3400RS quaternary pump, WPS-3000 TRS autosampler, TCC-3000RS column older, and PDA-3000RS detector. The analytical separation of compounds was achieved using a Hypersil Gold aQ C18 column (100 × 2.1 mm, 1.9 μm particle size), held at 30 °C, and at a constant flow of 0.3 mL/min with water–formic acid (90:10, *v*/*v*) (solvent A) and acetonitrile–formic acid (99.9:0.1, *v*/*v*) (solvent B). The gradient program of solvent A was as follows: 0–20 min from 98% to 30%; 20–24 min isocratic at 30%, then equilibration at the initial conditions for 9 min. The diode array detector was set at an acquisition range of 220–600 nm. The injection volume of the extracts (previously filtered at 0.22 µm) was 5 μL. Data were acquired and processed using Xcalibur v. 2 (Thermo Fisher Scientific, Waltham, MA, USA). The identification of the compounds was carried out by comparing the retention times and the spectral parameters of peaks with those of the standards. Specifically, rutin hydrate for semi-quantification of flavonoids; gallic acid, and syringic acid for semi-quantification of phenolic acids; malvidin-3-*O*-glucoside was used for semi-quantification of anthocyanins. Quantitative analysis was performed according to the external standard method based on calibration curves obtained by injecting different concentrations of standard solutions (R^2^ = 0.9972−0.9999). The results were expressed in mg of compound per kg of dry GP. All analyses were performed in triplicate. 

### 3.6. Cell Culture and Treatment

HT29 and SW480 human colon cancer cell lines were grown in the culture medium RPMI and DMEM, respectively. Cell lines were cultured in RPMI or DMEM in monolayer culture and supplemented with 10% FBS, 100 U/mL penicillin, 100 µg/mL streptomycin, and incubated at 37 °C in a humidified atmosphere containing 5% CO_2_ in the air. HT29 and SW480 were seeded in opportune culture plates (Becton Dickinson Labware, Franklin Lakes, NJ, USA) in a standard medium. After 24 h, the cells were stimulated with Aglianico GP gastrointestinal digested extract at increasing concentrations (25 μg/mL, 50 μg/mL, 80 μg/mL, and 100 μg/mL), and with a sample without Aglianico GP extract (containing 0 μg/mL of polyphenols) as CTR (Section 2.3). Thus, cells were incubated at 37 °C in an atmosphere of 5% CO_2_ in the air for 24 h, 48 h, and 72 h.

### 3.7. Cell Viability

Cell viability was evaluated by the 3-[4,5-dimethylthiazol-2-yl]-2,5 diphenyl tetrazolium bromide MTT test, according to Gigante et al. [18]. Briefly, all cells were treated with a range of concentrations of polyphenols starting from 0 (CTR), 25, 50, 80, and 100 μg/mL of Aglianico GP gastrointestinal digested extract for 24, 48, and 72 h. The assay is based on the assumption that the MTT tetrazolium salt reduction is due to the activities of mitochondrial dehydrogenases. After 24, 48, and 72 h of exposure in the culture medium, the MTT stock solution (5 mg/mL in medium) was added to each dish at a volume of one-tenth of the original culture volume and incubated for 2 h at 37 °C in humidified CO_2_. The medium was replaced with acidic isopropanol (0.1 N HCl absolute isopropanol). Formazan formation was spectrophotometrically monitored at 570 nm using an Evolution 60s UV-visible spectrophotometer (Thermo Fisher Scientific, Rodano, Italy). 

### 3.8. Apoptosis Analysis: Annexin V and Hoechst 33342 Assays

HT29 and SW480 cells were cultured as described above. Flow cytometry technology (Muse Cell Analyzer, Millipore, Darmstadt, Germany) was employed for quantitative analysis of live, early/late apoptotic, and dead cells based on the activation status of Annexin V. Cell marker (7-AAD), which was also used for dead cells. The cells were then processed following the user’s guide. Whole viable cells were also stained with Hoechst 33342 (Sigma Chemical Co., St. Louis, MO, USA) according to Mancini et al. The DNA fluorescence was measured using an Eclipse Ti2 microscope (Nikon Inc., Melville, NY, USA). Data acquisition was performed using NIS-Elements AR software. Three independent experiments were performed in triplicate and the results were indicated in the graphs as means ± SD.

### 3.9. Western Blotting

Western blotting was conducted according to Gigante et al. [18]. Briefly, the protein concentration of the protein extracts obtained by treating HT29 and SW480 cells with concentrations of 0 (CTR), 25, 50, 80, and 100 μg/mL of Aglianico GP gastrointestinal digested extract for 24 h was measured using the Bradford standard test (Bio-Rad, Milan, Italy). A total of 50 μg of protein extracts was loaded on prefabricated 4–12% polyacrylamide gel (Bio-Rad, Milan, Italy) for Western blot analysis. After the run the gel was transferred onto a nitrocellulose membrane and Bax (Cat. no #5023, Cell Signaling Technology, Danvers, MA, USA (CST), 1:1000), Bcl-2 (Cat. no #3498, Cell Signaling Technology, Danvers, MA, USA (CST), 1:1000), Caspase-3 (Cat. no #9662, Cell Signaling Technology, Danvers, MA, USA (CST), 1:1000), and Caspase-8 (Cat. no #9746, Cell Signaling Technology, Danvers, MA, USA (CST), 1:1000) were used as primary antibodies. Following overnight incubation, the membranes were incubated with horseradish peroxidase conjugated with anti-mouse or anti-rabbit IgG (Bio-Rad, Hercules, CA, USA); then, the proteins were detected by the chemiluminescence system (ECL). The signals were analyzed by laser densitometry with Chemi Doc System and Image Lab software (Bio-Rad Laboratories Inc., Hercules, CA, USA) and normalized against β-actin (Cat. no #4970, Cell Signaling Technology, Danvers, MA, USA (CST), 1:1000) expression.

### 3.10. Statistical Analysis

All data were expressed as means ± SD. Significant differences between the values of all parameters were determined at *p* < 0.05 The significance of the differences was analyzed by analysis of variance (ANOVA) followed by Tukey’s test of significant difference (HSD), Fisher’s minimum significant difference test (LSD) for multiple comparisons, and Dunnett’s post-test. The statistical analysis was performed by GraphPad Software (GraphPad Software, Inc., San Diego, CA, USA).

## 4. Conclusions

Notably, the main results of our work highlight the fact that the red winemaking Aglianico GP gastrointestinal digested extract represents a favorable source of polyphenolic compounds with antioxidant activity. Since in vitro gastrointestinal digestion is involved in most of the bioaccessibility of polyphenols, it was of relevant importance to investigate the effects of these extracts on cell proliferation and the pro-apoptotic effects on cancer cells. In fact, our experiments confirmed the anti-proliferative effect of the GP gastrointestinal digested extract (i.e., viability, proliferation, and apoptosis). This suggests that GP extract could be used in clinical practice for the treatment of colorectal cancer in the future. In conclusion, further studies will be conducted to determine the precise chemopreventive and chemotherapeutic molecular mechanisms underlying these beneficial actions.

## Figures and Tables

**Table 1:** Antioxidant activity and TPC of Aglianico GP gastrointestinal digested extract.

Determination	Aglianico GP Gastrointestinal Digested Extract
ABTS (µmol TE/g DW)	36.00 ± 0.24
DPPH (µmol TE/g DW)	15.18 ± 0.10
TPC (mg GAE/g DW)	5.01 ± 0.16

All values are means ± SD belonging to the three replicate measurements. Abbreviation: ABTS, 2,2′-azino-bis(3-ethylbenzothiazoline-6-sulfonic acid; DPPH, (2,2-diphenyl-1-picrylhydrazyl); GAE, gallic acid equivalent; TE, Trolox equivalent; TPC; total phenol content; DW, dry weight.

**Table 2:** Quantified sample content (mg/kg DW ± SD) of the main classes of phenolic compounds by the UHPLC-DAD analysis.

Classes	Compounds	mg/kg DW ± SD
**Anthocyanins**	Peonidin-3-glucoside	20.4 ± 1.1 ^d^
	Malvidin-3-*O*-glucoside	184.5 ± 19.3 ^a^
	Vitisin A	18.6 ± 0.4 ^d^
	Malvidin-3-*O*-acetylglucoside	43.5 ± 6.3 ^c^
	Malvidin-3-*O*-p-coumaroylglucoside	148.7 ± 1.7 ^b^
**Phenolic acid**	Gallic acid	189.2 ± 4.9 ^a^
	Syringic Acid	21.3 ± 2.3 ^d^
**Flavonoids**	Rutin hydrate	17.9 ± 1.5 ^d^
**Total**		644.0 ± 38.7

All values are means ± SD belonging to the three replicate measurements. Statistically significant means (*p* ≤ 0.05, one-way ANOVA and Fisher LSD test) are indicated by letters (^a^^–d^).

## Data Availability

Data are contained within the article.

## Supplementary Materials

The following supporting information can be downloaded at: https://www.mdpi.com/article/10.3390/molecules27206791/s1, Figure S1: Image of HT29 (A) and SW480 (B) cells, treated with increasing concentrations of Aglianico GP gastrointestinal digested extract compared to the control, stained with Hoechst 33342. Figure S2: Mitochondrial apoptosis signals by Aglianico GP gastrointestinal digested extract.
