# Supplementary material for: Anti-Proliferative and Pro-Apoptotic Effects of Digested Aglianico Grape Pomace Extract in Human Colorectal Cancer Cells
Source: Molecules. 2022 Oct 11;27(20):6791. doi: 10.3390/molecules27206791 (PMC9611208; doi:10.3390/molecules27206791)
Supplement: Supplementary file 1 [file molecules-27-06791-s001.zip › molecules-1916386-supplementary.pdf]

Supplementary Materials

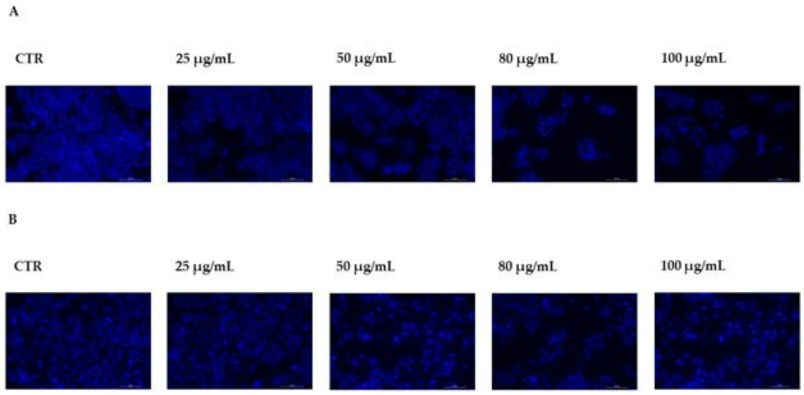

Figure S1. Image of HT29 (**A**) and SW480 (**B**) cells, treated with increasing concentrations of Aglianico GP gastrointestinal digested extract compared to the control, stained with Hoechst 33342.

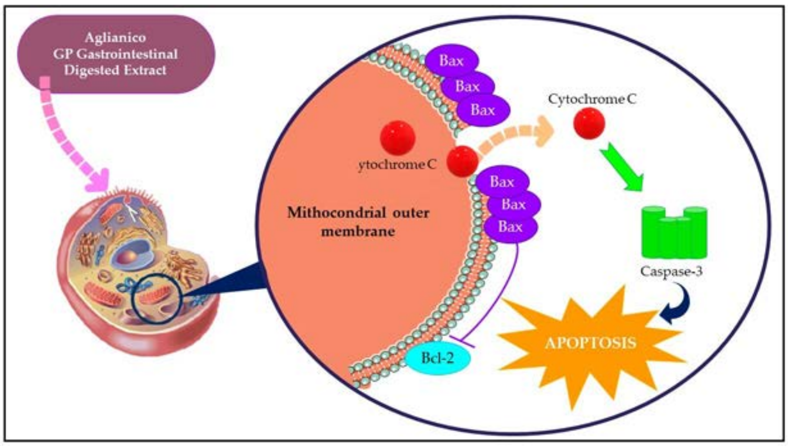

Figure S2. Mitochondrial apoptosis signals by Aglianico GP gastrointestinal digested extract.
